# Different Lower Limb Muscle MRI Patterns in Autosomal Dominant Titinopathies

**DOI:** 10.1111/ene.70348

**Published:** 2025-09-30

**Authors:** David Gómez‐Andrés, Laura Costa‐Comellas, Jordi Díaz‐Manera, Katrin Õunap, Mireia Álvarez‐Molinero, Gabriela Urcuyo, Marco Savarese, Francina Munell, Bjarne Udd

**Affiliations:** ^1^ Pediatric Neurology, Vall d'Hebron Institut de Recerca (VHIR), Hospital Universitari Vall d'Hebron, Vall d'Hebron Barcelona Hospital Campus, Department of Pediatrics Universitat Autònoma de Barcelona Barcelona Spain; ^2^ The John Walton Muscular Dystrophy Research Center Newcastle University, Newcastle upon Tyne NHS Trust Newcastle upon Tyne UK; ^3^ Neuromuscular Disorders Laboratory Institut de Recerca de l'Hospital de la Santa Creu i Sant Pau Barcelona Spain; ^4^ Centro de Investigación Biomédica en red en Enfermedades Raras (CIBERER) Barcelona Spain; ^5^ Department of Clinical Genetics, Genetics and Personalized Medicine Clinic Tartu University Hospital Tartu Estonia; ^6^ Department of Genetics and Personalized Medicine, Institute of Clinical Medicine, Faculty of Medicine University of Tartu Tartu Estonia; ^7^ Folkhalsan Research Center Helsinki University Helsinki Finland; ^8^ Tampere Neuromuscular Center University Hospital Tampere Finland

**Keywords:** hereditary myopathy with early respiratory failure, magnetic resonance imaging, muscle MRI, tibial muscular distrophy

## Abstract

**Background and Purpose:**

Titin is critical for sarcomere structure and function, and mutations in this gene cause titinopathies, a group of neuromuscular disorders. Muscle MRI is a key tool for diagnosing and understanding these conditions. This study aims to compare the muscle involvement patterns in two autosomal dominant, adult‐onset titinopathies: Tibial Muscular Dystrophy (TMD) and Hereditary Myopathy with Early Respiratory Failure (HMERF).

**Methods:**

In this multicenter, cross‐sectional study, lower limb MRI scans were analyzed for 17 patients with TMD and 15 with HMERF. Clinical and demographic data were collected from medical records. Muscle fat replacement was assessed using a modified Mercuri score for 30 muscles per patient. Two independent evaluators reviewed the images. Heatmaps were used to visualize asymmetry and patterns of fat replacement. Statistical analysis included Cliff's delta and random forests to distinguish muscle involvement between TMD and HMERF, and Spearman's rho to explore correlations between fat replacement and disease duration.

**Results:**

HMERF showed extensive, severe fat replacement, particularly in muscles like the semitendinosus, obturator externus, and gluteus minimus, with distinct intramuscular patterns. In contrast, TMD presented more localized fat replacement, primarily affecting the tibialis anterior and extensor digitorum longus. Both conditions exhibited mixed patterns of muscle replacement and preservation. Random forests confirmed differential muscle involvement, and fat replacement correlated with disease duration more strongly in HMERF than in TMD.

**Conclusions:**

This systematic MRI analysis reveals distinct muscle involvement patterns in TMD and HMERF, providing insights into the differential progression of these titinopathies.

## 1 Introduction

Titin stands as the largest known human protein. Titin is coded by *TTN*, the human gene with the most exons and the longest coding sequence [1]. In striated (both in cardiac and in skeletal) muscle, each titin molecule pairs with another antiparallel titin molecule within the sarcomere, creating a continuous elastic myofilament that spans the full length of the sarcomere and serves as a scaffold for multiple ligands [2]. Titin is a large, but also a complex protein that interacts with numerous proteins within the sarcomere. Titin contains an amino‐terminal Z‐disc region, two middle regions (the I‐band with the immunoglobulin‐like domains and the “PEVK” domain that are involved in the flexibility of titin, and the A‐band with multiple myosin and C‐protein binding sites), and a carboxy‐terminal M‐band region with a kinase domain and binding sites for obscurin, calpain3, and other proteins [3]. Moreover, in order to adapt to sarcomeres of different sizes and different mechanical properties, different isoforms of titin can be expressed due to differential splicing [4].

Titinopathies (skeletal muscle disorders due to mutations in TTN) are challenging for genetic diagnosis due to their complexity, and the size complicates functional studies and “in silico” pathogenic predictions. Titinopathies are a highly diverse group of clinical disorders, reflecting the complexity and multifunctional role of titin in the sarcomere. Therefore, additional helpful diagnostic tools are important, and muscle MRI has gained a central role for deep phenotyping of myopathies [3]. Some monoallelic TTN truncating variants can be associated with an autosomal dominant dilated cardiomyopathy with reduced, age‐dependent penetrance. Heterozygous non‐truncating variants also cause two previously established adult‐onset skeletal muscle disorders: (1) tibial muscular dystrophy (TMD; OMIM #600334) and (2) hereditary myopathy with early respiratory failure (HMERF; OMIM #603689). All reported TMD mutations are within the last exon 364, and HMERF is mostly caused by missense mutations in exon 344 that encodes the 119th fibronectin‐3 domain of A‐band titin [3]. Recently, earlier‐onset dominant titinopathies were reported as caused by intragenic deletions, but the systemic evaluation of muscle MRI findings is still pending [5].

TMD is a distal myopathy marked by ankle dorsiflexion weakness and an inability to walk on heels after age 35–40. Clinically, later involvement of knee flexors and glutei causes additional impairment [6, 7, 8]. Muscle MRI reveals selective fatty degeneration in anterior leg muscles, emphasizing its diagnostic utility [9]. TMD is particularly common in Finnish and Estonian populations due to the founder FINmaj mutation, but TMD also occurs in other populations with other mutations in exon 364 [3, 10]. HMERF is also a progressive disorder, typically starting between the ages of 30 and 50 with respiratory failure. This condition can also present with foot drop or frequent falls due to the early involvement of distal leg muscles [11]. It clinically progresses to involve axial muscles, proximal lower limbs, and upper limb muscles. Early involvement of semitendinosus has been reported as a typical finding in MRI [11, 12] although systematic description has not been performed yet.

Our objective is the description of lower limb involvement of TMD and HMERF in a multicenter study of patients whose MRI studies were retrospectively collected and systematically evaluated. We have compared semiquantitative involvement of the different muscles in the two dominant, adult‐onset titinopathies with the aim of studying shared features and differences.

## 2 Materials and Methods

We collected 32 lower limb muscle T1 or Dixon MR images of patients with an established diagnosis of adult‐onset dominant titinopathy (17 TMD patients and 15 HMERF patients) in a multicenter, cross‐sectional study, including evaluated patients from the participating centers in Finland, Estonia, and United Kingdom. We included images from all available patients with MR performed after 2005, with minimal quality to evaluate bilateral at least two of the three following regions (pelvis, thigh and lower leg) and with molecular confirmation of a pathogenic mutation in TTN. Genetic findings were reviewed to ensure the diagnosis. Age of onset and disease duration were retrospectively collected from medical records. All patients provided informed consent for their medical records and imaging data to be shared for research purposes. The study falls under the following approval provided by the Helsingin ja Uudenmaan sairaanhoitopiiri (HUS) research ethics committee: HUS/16896/2022.

Lower limb muscle MR images were reviewed and, if available, 30 muscles were systematically scored with a modified Mercuri score system [13]. Extensor digitorum longus and extensor hallucis longus were evaluated together. A normal muscle was scored by 0. A score of 1 for scattered small areas of increased density. If muscle was replaced by 30%–60%, we scored 2 and if the replacement implies 60%–90%, 3. A score of 4 was used for muscles with complete or nearly complete fat replacement. Each patient was evaluated by two independent reviewers (DGA and LCC). Discrepancies were discussed, and a final score was decided by consensus. Significant asymmetric involvement between right and left limb was also evaluated in each muscle.

Moreover, we observed a particular distribution of the fat replacement within some muscles in some patients. We later assessed systematically these signs of intramuscular fat replacement in each patient to describe frequency in each cohort and the relation with the global severity of muscle fat replacement.

Muscle pattern of fat replacement was represented by heatmaps for each form of titinopathy and the whole group of patients using the methodology described elsewhere [14, 15, 16]. In these heatmaps, we also represent the presence of asymmetry between left and right sides.

To evaluate the reproducibility and diagnostic utility of the imaging features identified in this study, we included an independent reviewer (G.U, a junior clinician with neuromuscular expertise who has only colaborated in the data analysis without previous access to full image dataset). She was blinded to the clinical diagnosis and provided with anonymized lower limb MRI datasets, the heatmaps and the images available in the paper and asked to classify each patient as either HMERF or TMD, based solely on imaging findings and the visual characteristics described in our analysis. The reviewer's classifications were compared to the reference diagnosis to calculate overall accuracy, and a confusion matrix was constructed to analyze misclassifications.

We estimated differences in the degree of fat replacement using Cliff's delta, an effect size statistics for comparison of ordinal values [17]. It represents the probability that a randomly chosen value from one group will be larger than a randomly chosen value from another group. Cliff's delta ranges from −1 to 1, where 0 indicates no difference, −1 indicates that all values in one group are smaller than in the other, and 1 indicates that all values in one group are larger than in the other. We also assessed multivariate pattern differences by means of random forests (ntree = 1000, mtry = 5) [18, 19]. Predictive mean matching was used for imputation of missing data [20] and mean decrease in Gini coefficient is used as a measure of variable importance. Out‐of‐the‐bag prediction accuracy was used as an indicator of the predictive capacity of the trained random forest, which was used as a proxy for multivariate differences.

We studied the correlation of fat replacement with disease duration within TMD and HMERF groups using Spearman's rho coefficient. 95% bias‐corrected and accelerated bootstrap intervals were calculated and represented by a Forest plot.

## 3 Results

Table 1 summarizes clinical variables and mutation of the included patients. In the HMERF cohort, individuals' ages span from 33 to 70 years, with the onset of the disease occurring between 25 and 53 years of age. Respiratory failure is the predominant symptom, although some also exhibit ankle dorsiflexion weakness. The duration of the disease varies from 0 to 25 years. On the other hand, TMD patients, aged between 38 and 71 years, typically present with ankle dorsiflexion weakness as their primary symptom between the ages of 35 and 45. In one instance, this symptom was only detected during a physical examination. Disease duration in TMD patients ranges from 0 to 29 years. All TMD patients carry the FINmaj mutation, whereas the mutations observed in the HMERF group are more diverse, with nine patients exhibiting p.C31712R, four patients showing a combination of p.P31732L linked with p.R34091W, and two patients presenting with p.A30143V mutation.

**TABLE 1:** Clinical description and mutation of each patient.

Patient	Sex	Age (years)	Onset (years)	Disease duration (years)	Symptoms at onset	Mutation (NM_001267550.1)^a^	Exon
**HMERF patients**							
HMERF1	F	40	35	5	resp failure	p.P31732L + *p*.R34091W = dominant allele	344 + 359
HMERF2	F	45	38	7	resp failure	p.P31732L + *p*.R34091W = dominant allele	344 + 359
HMERF3	M	41	32	9	resp failure	p.C31712R	344
HMERF4	F	43	35	8	resp failure	p.C31712R	344
HMERF5	F	70	45	25	resp failure	p.A30143V	344
HMERF6	F	66	53	12	resp failure	p.A30143V	344
HMERF7	M	47	42	5	resp failure	p.P31732L + *p*.R34091W = dominant allele	344 + 359
HMERF8	F	66	51	15	resp failure	p.P31732L + *p*.R34091W = dominant allele	344 + 359
HMERF9	F	33	25	8	Ankle dorsiflexion weakness	p.C31712R	344
HMERF10	M	38	37	1	Ankle dorsiflexion weakness	p.C31712R	344
HMERF11	F	44	36	8	resp failure	p.C31712R	344
HMERF12	M	43	30	13	Ankle dorsiflexion weakness	p.C31712R	344
HMERF13	F	45	45	0	Ankle dorsiflexion weakness	p.C31712R	344
HMERF14	M	48	46	2	Ankle dorsiflexion weakness	p.C31712R	344
HMERF15	M	37	27	10	Ankle dorsiflexion weakness	p.C31712R	344
**TMD patients**							
TMD1	F	46	35	11	Ankle dorsiflexion weakness	FINmaj	364
TMD2	F	71	45	26	Ankle dorsiflexion weakness	FINmaj	364
TMD3	F	62	37	25	Ankle dorsiflexion weakness	FINmaj	364
TMD4	F	69	41	28	Ankle dorsiflexion weakness	FINmaj	364
TMD5	M	64	35	29	Ankle dorsiflexion weakness	FINmaj	364
TMD6	F	57	37	20	Ankle dorsiflexion weakness	FINmaj	364
TMD7	M	56	38	18	Ankle dorsiflexion weakness	FINmaj	364
TMD8	M	57	40	17	Ankle dorsiflexion weakness	FINmaj	364
TMD9	F	45	35	10	Ankle dorsiflexion weakness	FINmaj	364
TMD10	F	39	35	4	Ankle dorsiflexion weakness	FINmaj	364
TMD11	F	45	40	5	Ankle dorsiflexion weakness	FINmaj	364
TMD12	M	60	42	18	Ankle dorsiflexion weakness	FINmaj	364
TMD13	M	63	37	26	Ankle dorsiflexion weakness	FINmaj	364
TMD14	M	54	40	14	Ankle dorsiflexion weakness	FINmaj	364
TMD15	M	61	42	19	Ankle dorsiflexion weakness	FINmaj	364
TMD16	M	38	38	0	Subjectively asymptomatic	FINmaj	364
TMD17	M	69	40	29	Ankle dorsiflexion weakness	FINmaj	364

^a^ FINmaj = p.35927‐35930 delinsVKEK.

**FIGURE 1:**
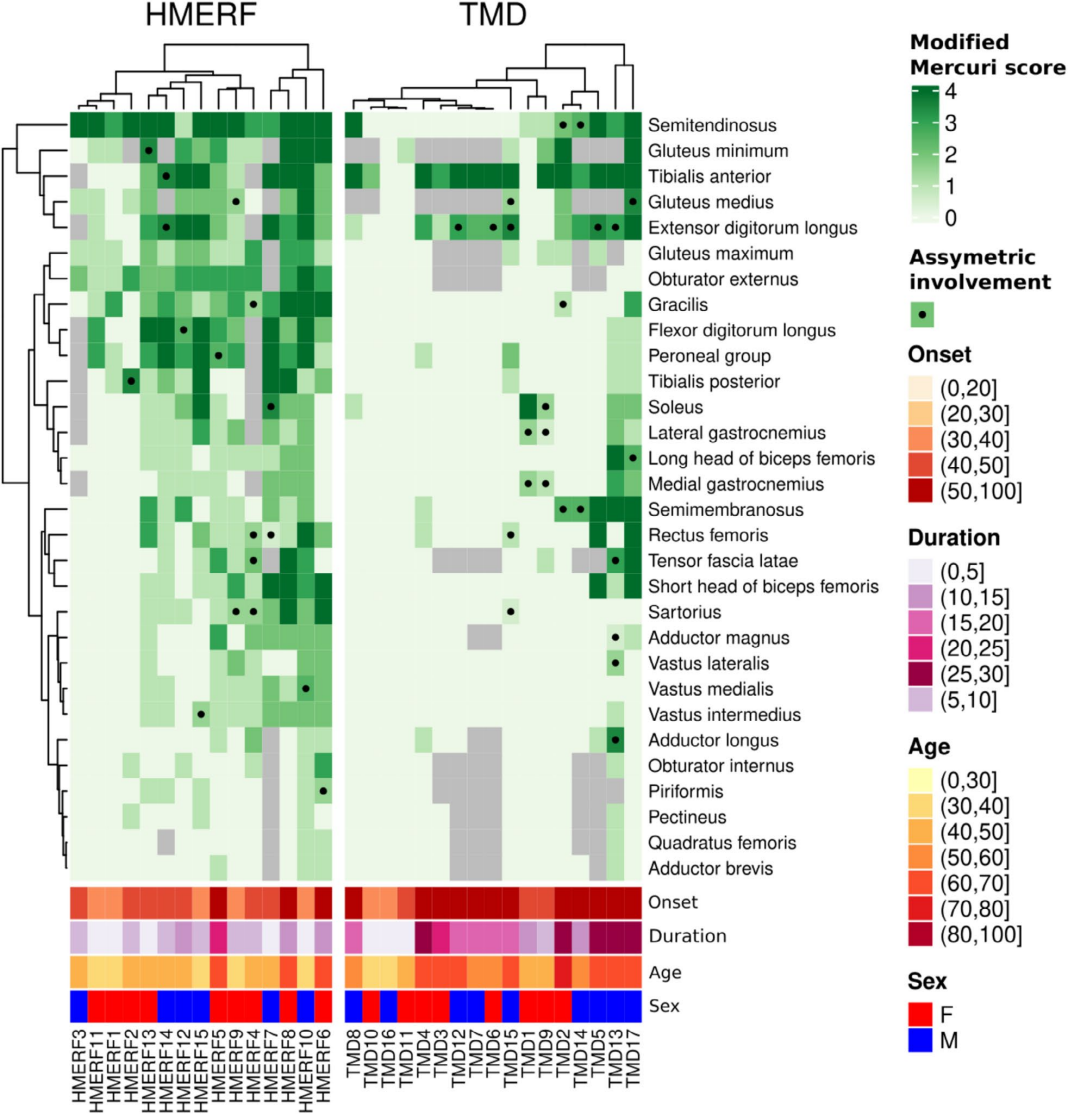
Heatmaps illustrating fat replacement pattern in HMERF (*left*) and TMD (*right*) patients. Fat replacement for every muscle in every patient is represented. The darker the corresponding square is, the higher fat replacement this muscle has. Asymmetry in fat replacement is represented by a point. Muscles are ordered according to how similar fat replacement is along the two groups of patients. Dendrogram in the left represents this hierarchical classification. Similarity between patients is shown in two dendrograms, one for HMERF patients and the other for TMD patients. In the bottom of the figure, patients' features are shown. Notice the differences in fat replacement profiles of the two titinopathies.

**FIGURE 2:**
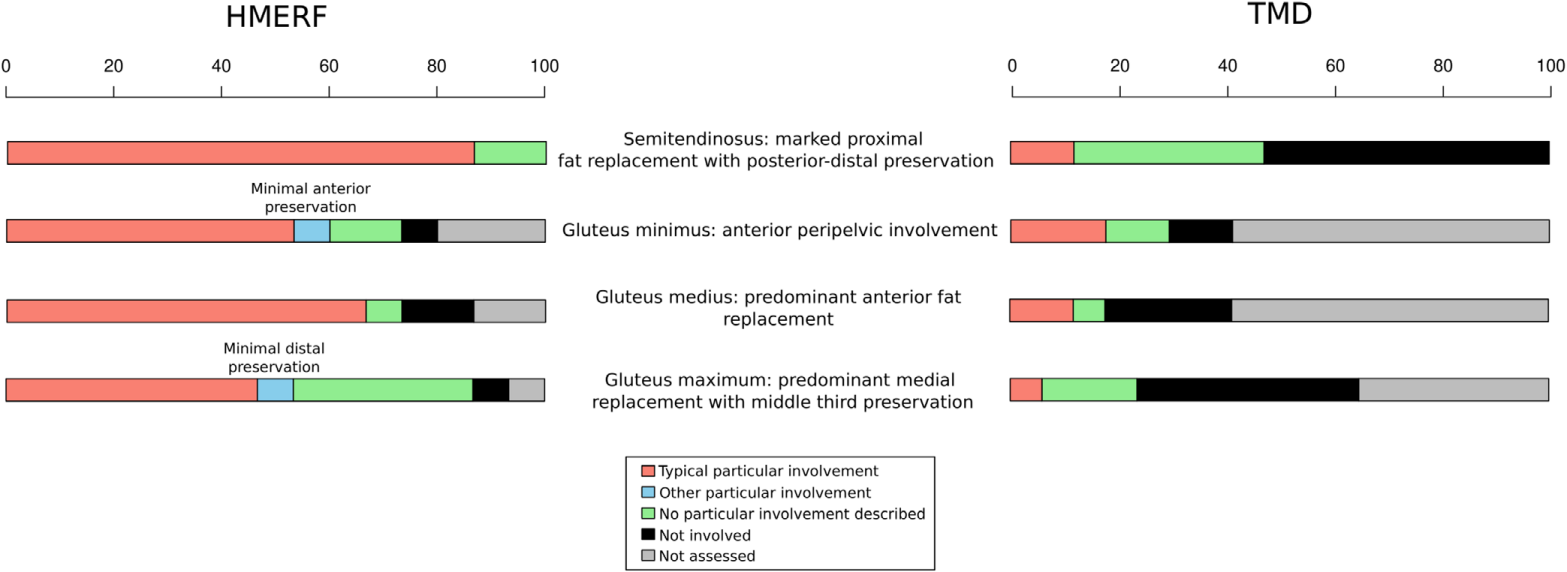
Barplot showing the frequency of particular intramuscular involvement in semitendinosus, gluteus maximus, medium and minimum.

In Figure 1, we present muscle involvement in HMERF and TMD patients using combined heatmaps. HMERF patients show higher degrees of muscle fat replacement and a more dispersed involvement than TMD patients. Figure 2 shows the presence of particular muscle signs occurring due to particular muscle replacement in specific muscles.

**FIGURE 3:**
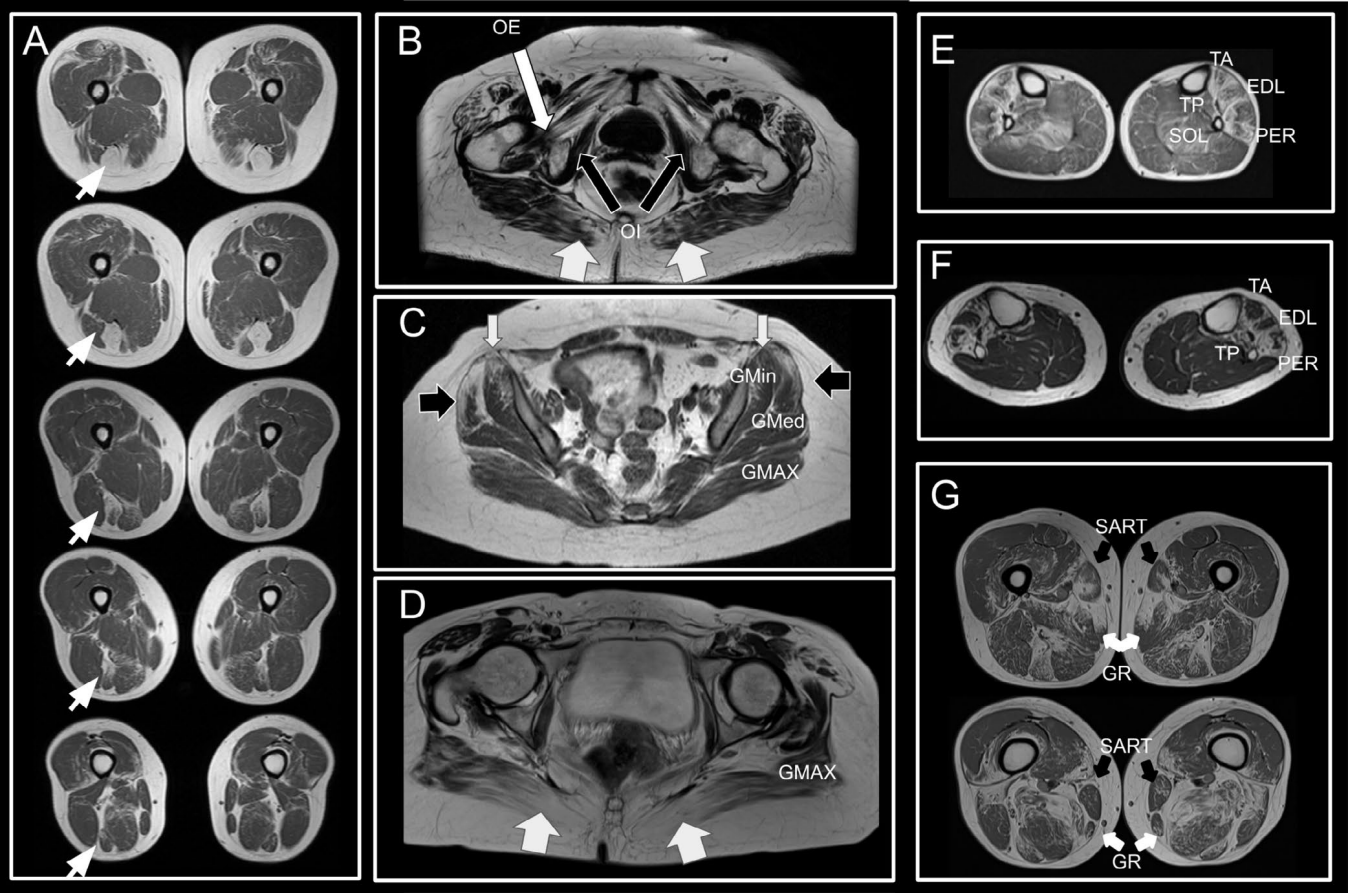
Selected MR images from different patients with HMERF. (A) Axial thigh images from proximal to distal are shown to demonstrate the typical involvement of semitendinosus muscle (white arrow in the left leg) in the disease. Complete fat replacement occurs in the proximal part of the muscle, whereas distally and posteriorly, muscle bulk is preserved. (B) Lower axial pelvic MR image (IDEAL) that shows the preservation of the obturator internus muscle in comparison to the obturator externus. Notice also the involvement of the medial part of gluteus maximus. (C) High axial pelvic view to glutei muscles. Notice the medial and anterior involvement of gluteus minimus (white arrows) and the anterior involvement of gluteus medius (black arrows). (D) Typical gluteus maximal involvement with severe medial involvement and preservation of the middle part. (E, F) Severe involvement of anterior compartment in two patients. Notice the heterogeneous intramuscular involvement of muscles. (G) Thigh images of the same patient. Notice the distal preservation of gracilis and sartorius in comparison to proximal involvement.

In HMERF patients (Figure 1‐left‐, Figure S1 and Figure 3), fat replacement profile is featured by early involvement of semitendinosus with a particular intramuscular fat replacement pattern (present in 13 out of 15 patients). Fat replacement predominantly occurs in the proximal part of the muscle, leaving a small area of muscle preservation in the distal posterior part of the muscle (Figures 2 and 3). Obturator externus is also systematically involved and comparison with obturator internus may be a guiding sign for diagnosis (Figures 2 and 3A). Gluteus minimus and medius are also muscles which are commonly involved in HMERF patients. Fat replacement in gluteus minimus is predominantly occurring in the anterior part of the muscle and along the pelvic insertion (Figure 3B). Fat replacement in gluteus medius is also significantly skewed in the anterior half of the muscle (Figure 3C). Gluteus maximus tends to have lower fat replacement scores, but it is also commonly involved showing a particular intramuscular fat replacement pattern consisting in a predominant medial involvement with a selective preservation of the middle third of the muscle (Figure 3D). Anterior leg compartment (tibialis anterior, extensor digitorum longus and flexor digitorum longus) and peroneal group are also muscles that are usually involved in these patients (Figure 3E), although any single pattern cannot be defined for these muscles. Fat replacement is often non‐homogeneous and muscles tend to have well‐preserved areas and areas of severe involvement if the muscle is not completely involved. Tibialis posterior is significantly involved in some patients. Gracilis muscle is also frequently involved and although a common pattern of intramuscular fat replacement was not possible to define, replacement was not homogeneously distributed in 7 out of 13 patients with gracilis involvement (Figure 3G). Sartorius is less involved than gracilis, but as with gracilis, replacement was not homogeneously distributed in 4 out of 10 patients who show involvement in this muscle (Figure 3G). Adductor longus and brevis, obturator internus, piriformis, pectineus and quadratus femoris are affected only in a mild form and few patients.

**FIGURE 4:**
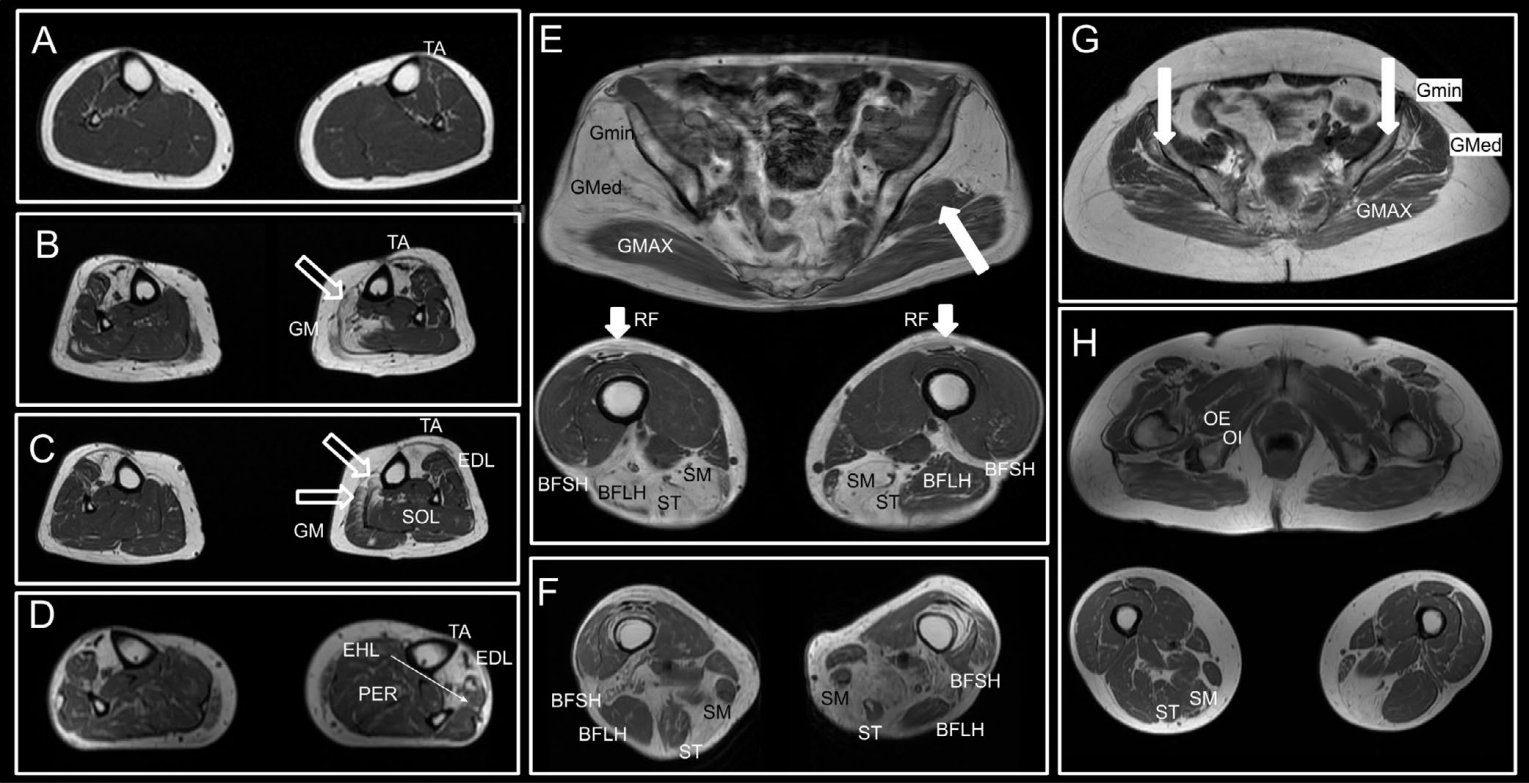
Selected MR images from different patients with TMD. (A–D) Axial images from leg muscles. Tibialis anterior (TA) is early involved, even in patients with minimal fat replacement (A). Generally tibialis anterior is severely involved (B–D). Extensor digitorum longus (EDL) is assymetrically involved. Some patients have assymetric and non‐homogeneous involvement of soleus (SOL) and gastrocnemius (GM). Peroneal muscles are mostly preserved (E–H). Axial images from pelvis and thigh of four different patients. Notice the asymmetry and very selective involvement of some muscles. The involvement of gluteus minimus (Gmin) that could be mild (G) or severe (E). Gluteus medium (GMed) could also be involved (E). Semimembranosus (SM), semitendinosus (ST) and biceps femoris (BFSH‐short head and BFLH—long head) are the other most commonly involved muscle outside the leg. Some patients may have severe atrophy of rectus femoris (RF). Obturator externus (OE) and internus (OI) are preserved muscles (H‐pelvis).

TMD patients (Figure 1‐right‐, Figure S2 and Figure 4) show a more restrictive fat replacement pattern with early and significant involvement of tibialis anterior and extensor digitorum longus (typically asymmetric—Figure 4A–D). Muscles of the posterior leg compartment are also involved in some cases, and as well in asymmetric pattern (Figure 4B,C). Fat replacement in these cases is also distributed in non‐homogenous patterns with some areas of the muscle severely involved and other areas with preservation. Some patients may have involvement of gluteus medius and minimus, semitendinosus and semimembranosus (sometimes asymmetric), and in some severe cases involvement of rectus femoris (4 out of 13 patients) with significant atrophy (one patients shows an asymmetric pattern) or of biceps femoris can occur.

The independent reviewer correctly classified 31 out of 32 cases, yielding an overall accuracy of 96%. Misclassifications occurred in a severe case of TMD which was misclassified as HMERF.

**FIGURE 5:**
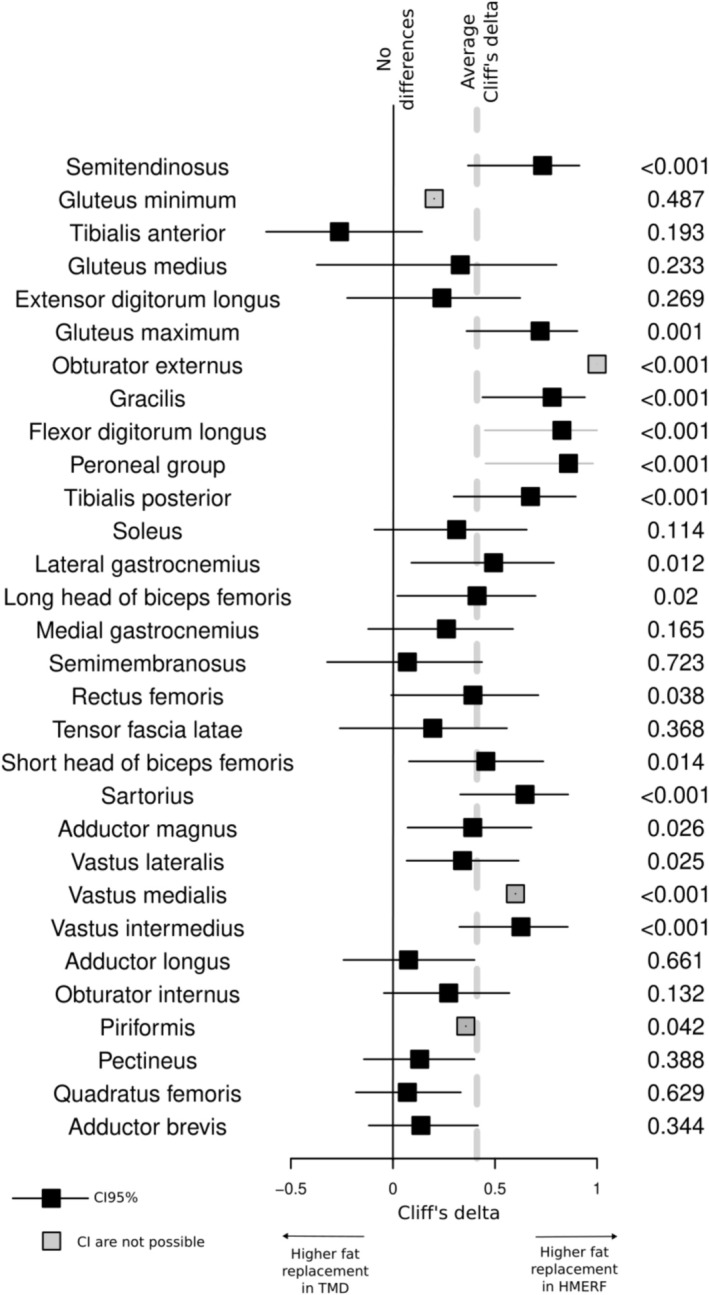
Forest plots showing the differences between HMERF and TMD in the fat replacement score for every assessed muscle. Square symbols represent the point estimates of Cliff's delta (Effect size statistics for ordinal variable comparison). Horizontal lines represent 95% confidence. Cliff's delta of 0 implies no difference (vertical black line). A Cliff's delta of 1 implies that the score for any HMERF patients would be higher than any TMD patient. A negative Cliff's delta would mean the opposite. Notice that the average delta is shown by a gray dotted line. *p* value for Wilcoxon's sign comparison is shown on the right. In some muscles, indicated with a gray color, calculation of 95% CI for the Cliff's delta was not possible.

HMERF and TMD have a differential pattern in fat replacement. We confirmed this by using random forests that illustrate a differential multivariate pattern (out‐of‐bag estimate of error rate = 6.25%). Important muscles for the classification are obturator externus, flexor digitorum longus, gracilis and semitendinosus. We also studied the differences in a univariate comparison in every assessed muscle (Figure 5). As previously indicated, HMERF patients tend to show higher overall replacement scores. Particularly, semitendinosus, obturator externus, peroneal group and flexor digitorum longus are more involved in HMERF. No muscle shows significantly higher scores in TMD, but tibialis anterior shows a tendency and Cliff's delta below the average of the muscles. The combined hierarchical analysis of the two diseases (dendrogram of the left part of Figure 1) allows the assessment of shared and differential features in fat replacement patterns in the diseases. Although there is a clear difference in degree and intramuscular fat replacement pattern between the two disorders, some muscles tend to be involved in both titinopathies: semitendinosus, glutei muscles and anterior leg compartment (tibialis anterior and extensor digitorum longus). Other muscles are well‐preserved in most of the patients, such as obturator internus, piriformis, pectineus, quadratus femoris and adductor brevis.

**FIGURE 6:**
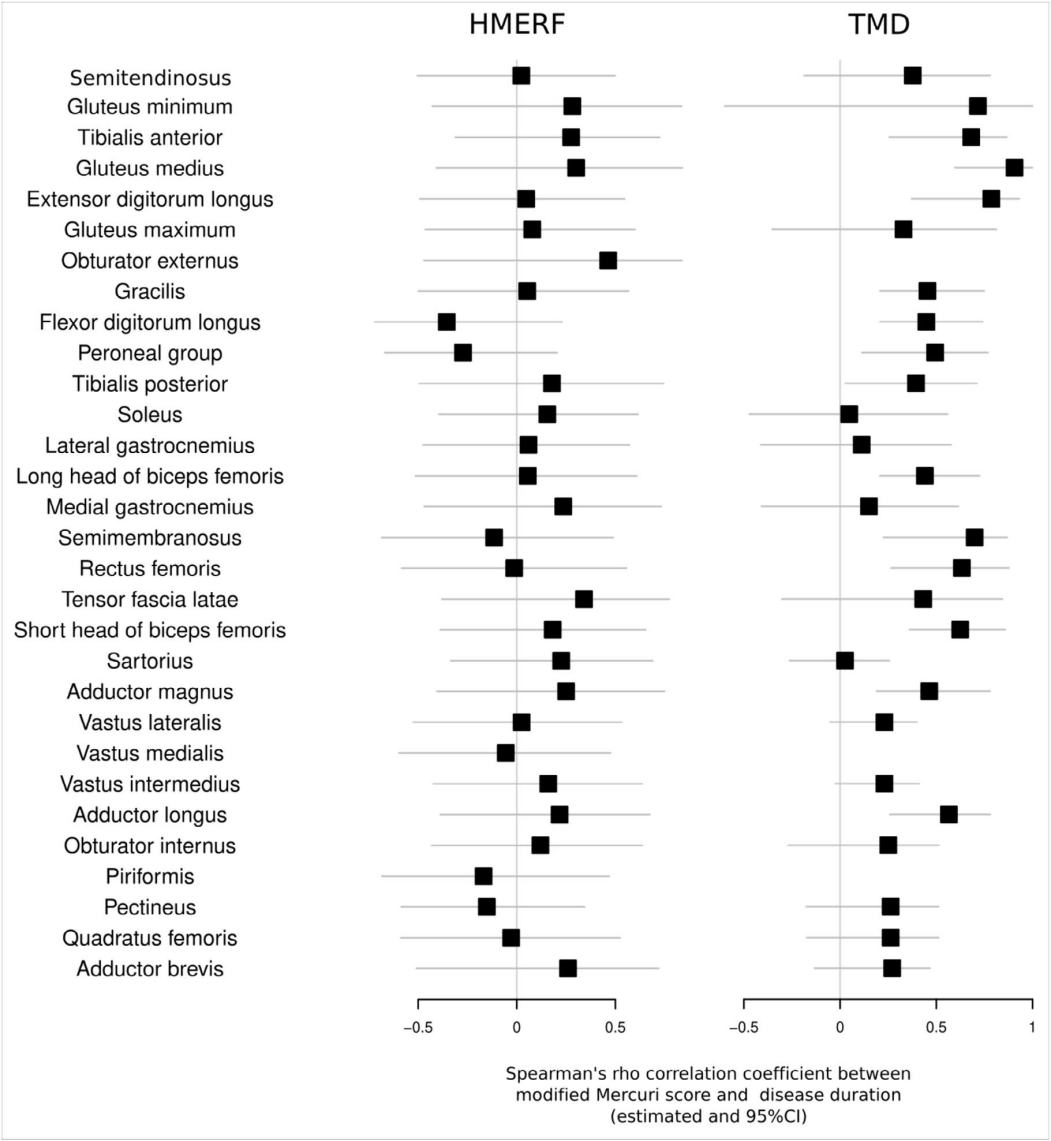
Forest plots showing the correlation between each muscle fat replacement score and disease duration in HMERF (*left*) and TMD (*right*). Square symbols represent the point estimates of Spearman's rho correlation coefficient between modified Mercuri score and disease duration. Horizontal lines represent 95% confidence. If horizontal lines do cross zero‐line (no correlation), no significant correlation should be interpreted.

The different correlations between fat replacement and disease duration were also found to be dissimilar between HMERF and TMD (Figure 6). Fat replacement scores for muscles in HMERF do not show any significant correlation with disease duration, whereas TMD patients show a positive correlation in a number of muscles including tibialis anterior, gluteus medius and extensor digitorum longus. This indicates that patients with a longer disease duration also show a higher degree of fat replacement.

## 4 Discussion

This study provides a systematic evaluation of lower limb muscle MRI in two dominant adult‐onset titinopathies—HMERF and TMD—highlighting both shared and distinct features of muscle involvement. In contrast to previous studies, which have largely offered descriptive narrative accounts [9, 11, 12], we applied a structured semiquantitative analysis across 30 muscles, incorporating heatmap visualizations, statistical comparisons, and correlation with disease duration. Our findings confirm overall differences in the extent and distribution of fat replacement between the two disorders, and identify several imaging features—particularly intramuscular patterns and asymmetries—that may aid in their differentiation. Furthermore, we observed that in TMD, but not in HMERF, fat replacement shows a measurable relationship with disease duration, suggesting differences in disease progression dynamics.

HMERF patients show broader and more extensive muscle involvement compared to those with TMD. Certain muscles are consistently and significantly affected in HMERF—particularly the semitendinosus and obturator externus, the latter being more involved than the obturator internus. Notably, all HMERF patients in our cohort exhibited combined involvement of these two muscles, making this a potentially distinctive diagnostic marker. Additionally, the semitendinosus and gluteal muscles displayed characteristic intramuscular fat replacement patterns, which may serve as further diagnostic clues. In TMD, while the most prominent finding remains the involvement of anterior lower leg muscles, our analysis also revealed frequent proximal involvement, especially of the gluteus minimus and medius. We also observed marked asymmetry in fat replacement, particularly in proximal thigh muscles and the extensor digitorum longus. In more advanced cases, significant atrophy of the rectus femoris and biceps femoris was also present.

Although intramuscular fat replacements in particular muscles do not show an identifiable common distribution in the whole spectrum of these two disorders, there are important shared features between TMD and HMERF: generally, muscles tend to be affected in a non‐homogeneous way with areas in the muscle that are totally replaced while others are absolutely preserved. Some findings may be very evocative for a particular diagnosis such as the very particular pattern of semitendinosus involvement in HMERF, but the intramuscular pattern of severe involved versus preserved parts of muscles could be present in other muscles and should prompt a suspicion for titinopathy. Mechanistically, several hypotheses may explain this phenomenon. One possibility is the differential expression of titin or its interacting partners across muscles or within specific regions of a muscle [21, 22, 23]. Variability in titin isoform expression or differences in posttranslational processing may also contribute [24, 25]. Additionally, heterogeneous biomechanical strain across muscle regions could play a key role in shaping these intramuscular fat replacement pattern*s*.

Although a relationship between fat replacement and disease duration was clearly demonstrated in TMD, no individual muscle in HMERF showed a statistically significant correlation. This could be attributed to difficulties in precisely identifying the onset of symptoms in HMERF or to a more rapid and possibly nonlinear disease progression, with early and abrupt widespread muscle involvement followed by a stability plateau.

Regarding differential diagnostic implications (Figures S4 and S5), HMERF could clinically overlap with late‐onset Pompe disease. However, the tibialis anterior and other distal leg muscles are usually spared in Pompe, semitendinosus is involved only in very advanced stages, and obturator externus and internus are usually equally affected [26]. Other differential diagnostic alternatives are the myofibrillar myopathies. Disorders with early involvement of semitendinosus are rare [27, 28]. Desminopathies frequently show earlier involvement of peroneus, triceps surae, adductor brevis and longus, gracilis, and sartorius, muscles that are only involved in later stages of HMERF [27, 29]. Filaminopathies typically have early involvement of the complete posterior compartment of the thigh (not restricted to semitendinosus) with a characteristic intramuscular reticular distribution, so extremely different from the HMERF intramuscular phenotype [30]. GNE‐related myopathy also overlaps with HMERF due to semitendinosus and tibialis posterior involvement, but in GNE‐related myopathy, the thigh posterior compartment involvement expands to more muscles, quadriceps sparing is more extreme, glutei involvement tends to occur late, posterior lower leg muscles are involved earlier, and semitendinosus intramuscular fat distribution is different [31].

Clinical diagnosis of TMD is usually easy in populations with the common FINmaj presence, but in other countries, differentiating TMD from other distal myopathies and CMTs has been more challenging in the past. Some distal myopathies show a significant imaging overlap [32]. Welander distal myopathy shows a different clinical phenotype at onset (early upper limb involvement) [33] and particular differences have been described elsewhere (predominant involvement of the posterior lower limb compartment) [9]. Nebulin‐distal myopathy shares with TMD the severe involvement of the anterior lower leg compartment, but in contrast, there is marked preservation of thigh muscles and gastrocnemius with early soleus involvement [34, 35].

Our study has some limitations. First, it is a retrospective collection of muscle MR imaging at different stages of disease evolution and from different centers that use somewhat different muscle imaging protocols. Although a limitation, this contributed to collecting a significant number of patients with ultrarare disorders and to having information along the disease evolution and severity spectrum. Second, some muscles that may be important for the delineation of the phenotype, such as obturator externus, are small, and scoring of fat replacement was difficult in some cases. Third, due to availability reasons, we limited the analysis to lower limbs while we think that whole‐body involvement could be interesting, in particular for HMERF patients.

Our structured, semiquantitative approach to muscle MRI analysis—combined with the use of heatmaps and pattern‐based comparisons—could be a base for solid, fast implementation of automated image analysis workflows in the future. The consistent and region‐specific patterns we observed, particularly the intramuscular features in HMERF, suggest that deep learning models could be trained to identify these phenotypes with high accuracy. This is also supported by the high accuracy of neuromuscular clinicians in muscle MRI‐based classification for differentiating these TTN‐related myopathies, indicating that the features described may be reproducible when applied by other experienced readers [36].

Another relevant aspect for discussion is the comparison of MRI phenotypes among HMERF, TMD, and congenital titinopathies [36, 37, 38]. Despite clinical differences, these conditions consistently exhibit a characteristic pattern of intramuscular fat replacement, with some muscles or some parts of the muscles showing severe involvement while others are relatively preserved. This shared feature suggests common underlying mechanisms linked to titin mutations. However, significant differences exist in the pattern of affected muscles. HMERF and TMD show distinct fat replacement patterns compared between them and to congenital titinopathies, which are characterized by greater heterogeneity due to their association with a broader range of TTN mutations. The semitendinosus muscle is frequently affected in HMERF but shows variable involvement in congenital titinopathies and is only affected in a subset of TMD cases, as demonstrated in our study. These particular distributions of muscle involvement highlight the influence of mutation type and location on muscle involvement patterns in titinopathies [25], emphasizing the value of systematic MRI analysis in larger case series to clarify correlations between clinical phenotype, imaging phenotype, and genotype. In conclusion, we have systematically defined the MR imaging pattern for HMERF and TMD in the lower limbs. We also detect some common signs that could guide the diagnosis and sharing features that may be present in other titinopathies, such as the striking intramuscular fat replacement distribution or the asymmetry in muscle involvement. We have also demonstrated a clear relationship of disease duration with fat replacement in some muscles in TMD.

## Author Contributions

**David Gómez‐Andrés:** conceptualization, investigation, funding acquisition, writing – original draft, methodology, validation, visualization, writing – review and editing, software, formal analysis, project administration, data curation, supervision, resources. **Laura Costa‐Comellas:** conceptualization, investigation, methodology, validation, visualization, data curation. **Jordi Díaz‐Manera:** conceptualization, investigation, methodology, validation, visualization, resources, funding acquisition, writing – review and editing. **Katrin Õunap:** conceptualization, investigation, funding acquisition, methodology, validation, visualization, resources, writing – review and editing. **Mireia Álvarez‐Molinero:** writing – review and editing, investigation. **Gabriela Urcuyo:** investigation, writing – review and editing. **Marco Savarese:** investigation, conceptualization, funding acquisition, writing – review and editing, methodology, resources, validation. **Francina Munell:** conceptualization, investigation, funding acquisition, methodology, validation, writing – review and editing, resources, project administration, supervision. **Bjarne Udd:** conceptualization, investigation, funding acquisition, writing – review and editing, project administration, resources, supervision.

## Conflicts of Interest

The authors declare no conflicts of interest.

## Supporting information


**Figure S1:** Heatmap illustrating fat replacement pattern in HMERF patients.
**Figure S2:** Heatmap illustrating fat replacement pattern in TMD patients.
**Figure S3:** Bar plot showing the top 10 most important muscles in the discrimination between HMERF and TMD.
**Figure S4:** Flowchart for the differential diagnosis of HMERF and related myopathies.
**Figure S5:** Patterns of muscle involvement in distal myopathies which are a diffential diagnosis for tibial muscular dystrophy.

## Data Availability

The data that support the findings of this study are openly available in Figshare at https://doi.org/10.6084/m9.figshare.25461628.v1.
