# Supplementary material for: Different Lower Limb Muscle MRI Patterns in Autosomal Dominant Titinopathies
Source: Eur J Neurol. 2025 Sep 30;32(10):e70348. doi: 10.1111/ene.70348 (PMC12481456; doi:10.1111/ene.70348)
Supplement: Supplementary file 1 — Figure S1: Heatmap illustrating fat replacement pattern in HMERF patients. Figure S2: Heatmap illustrating fat replacement pattern in TMD patients. Figure S3: Bar plot showing the top 10 most important muscles in the discrimination between HMERF and TMD. Figure S4: Flowchart for the differential diagnosis of HMERF and related myopathies. Figure S5: Patterns of muscle involvement in distal myopathies which are a diffential diagnosis for tibial muscular dystrophy. [file ENE-32-e70348-s001.docx]

**
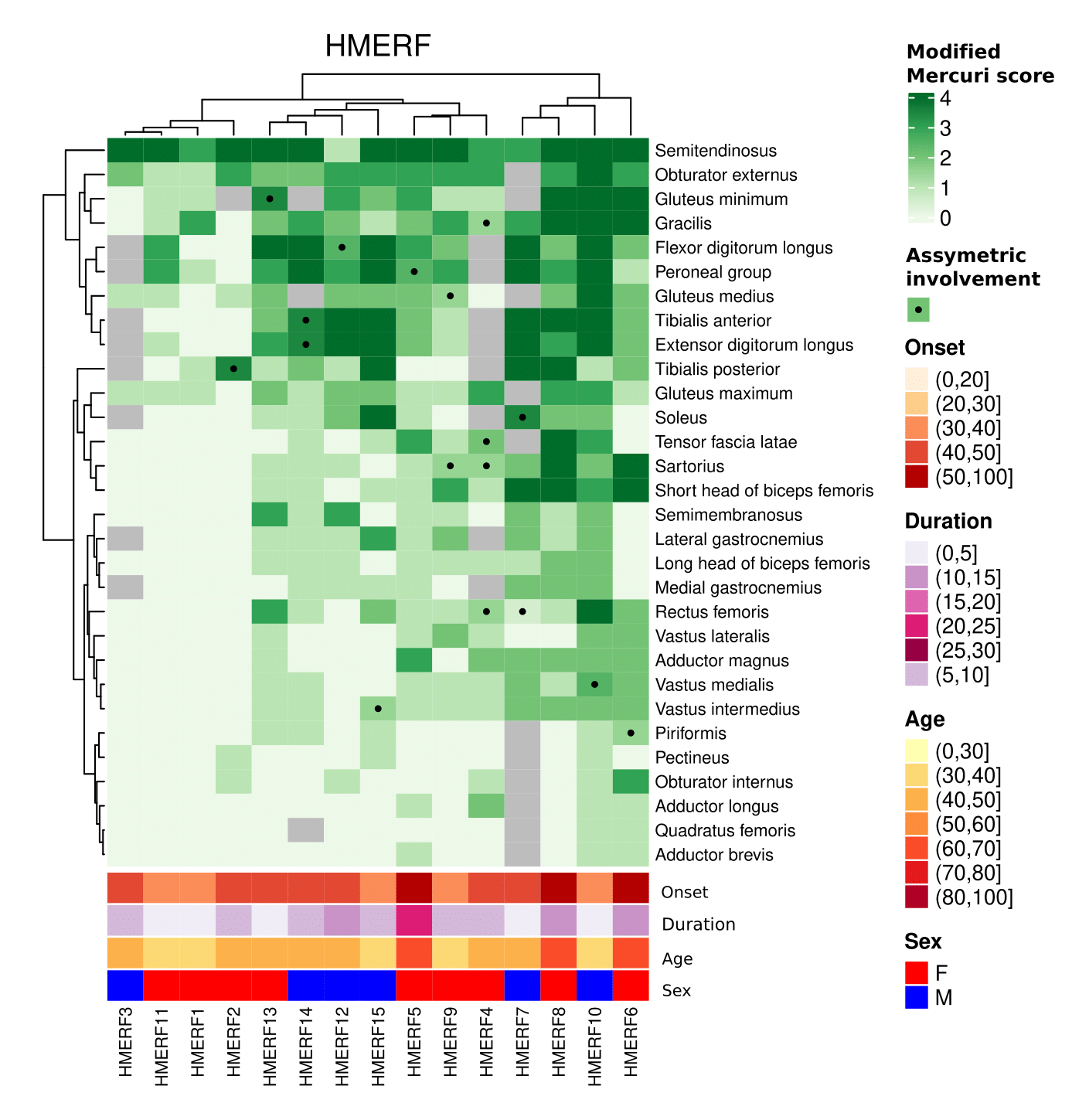
**

**Supplementary figure 1. Heatmap illustrating fat replacement pattern in HMERF patients. Fat replacement for every muscle in every patient is represented**. The darker the corresponding square is, the higher fat replacement this muscle has. Asymmetry in fat replacement is represented by a point. Muscles are ordered according to how similar fat replacement is along the HMERF patients. Dendrogram in the left represents this hierarchical classification. Similarity between patients is shown by the dendrogram above. In the bottom of the figure, patients’ features are shown.


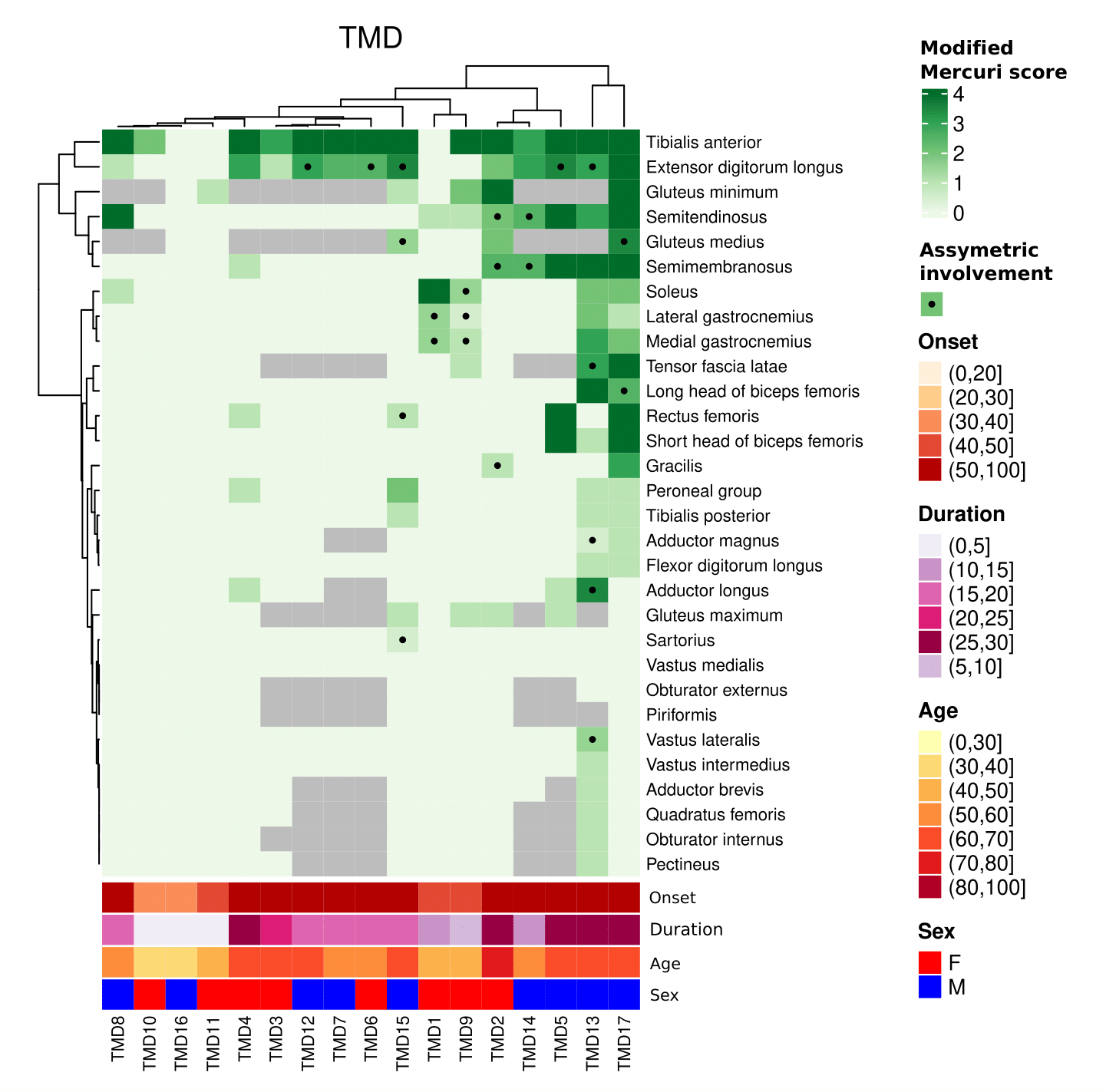


**Supplementary figure 2.**. **Heatmap illustrating fat replacement pattern in TMD patients.** Fat replacement for every muscle in every patient is represented. The darker the corresponding square is, the higher fat replacement this muscle has. Asymmetry in fat replacement is represented by a point. Muscles are ordered according to how similar fat replacement is along the TMD patients. Dendrogram in the left represents this hierarchical classification. Similarity between patients is shown by the dendrogram above. In the bottom of the figure, patients’ features are shown.

**
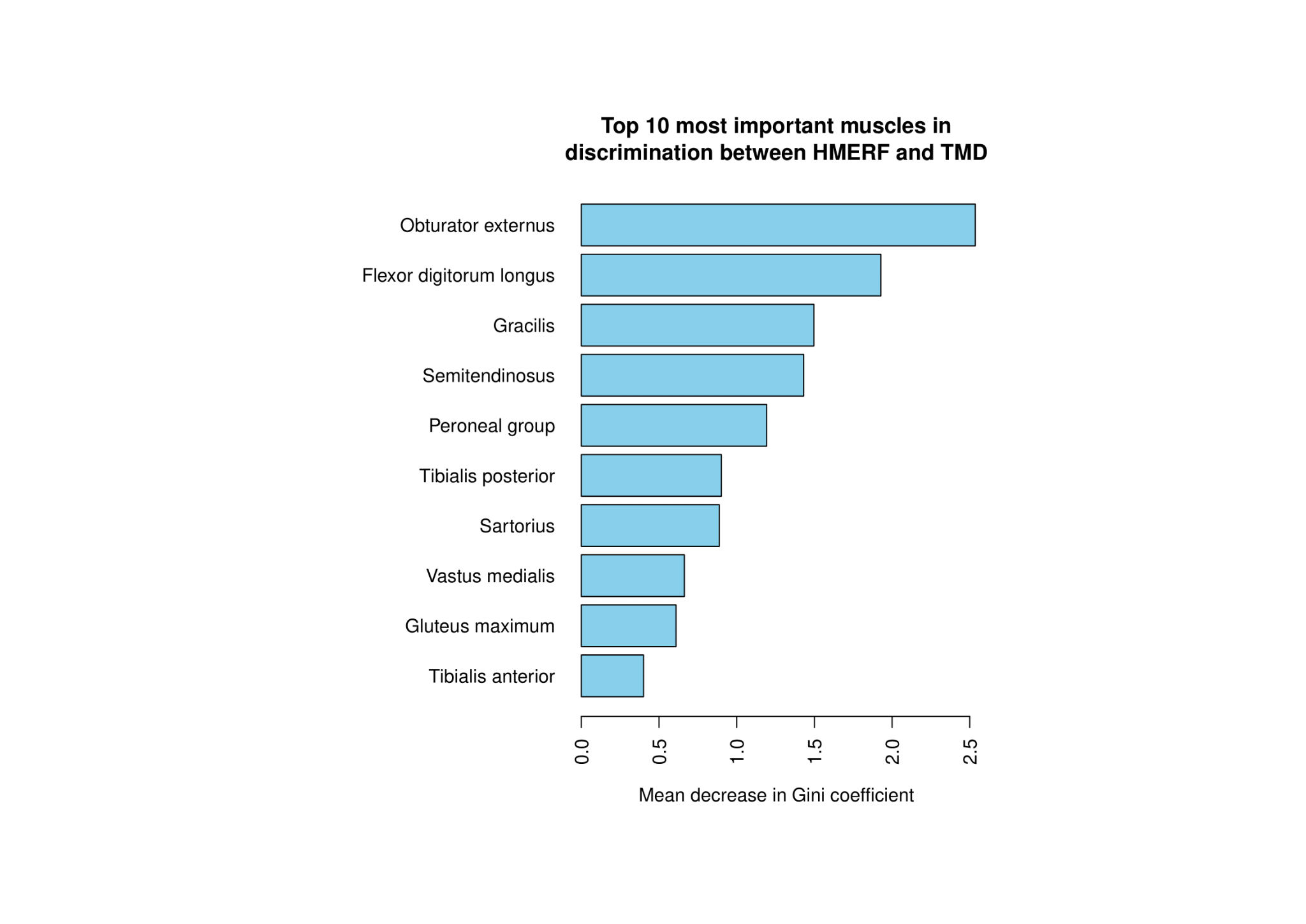
**

**Supplementary figure 3**. **Bar plot showing the top 10 most important muscles in the discrimination between HMERF and TMD.** Mean decrease in Gini coefficient was used for measuring variable importance in the random forest prediction. The higher the mean decrease in Gini coefficient is, the more weight this muscle has in the prediction.

**
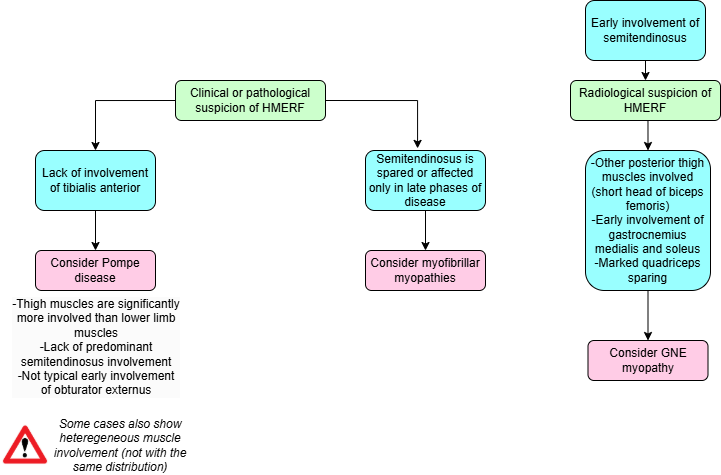
**

**Supplementary figure 4. Flowchart for the differential diagnosis of HMERF and related myopathies.** The diagram provides a structured approach for distinguishing HMERF from other muscle disorders based on clinical, pathological, and radiological findings. The left pathway highlights key features associated with Pompe disease, including the absence of tibialis anterior involvement and predominance of thigh muscle involvement. Intramuscular fat distribution within the muscles can be a misleading finding, as it can show similar behaviour to HMERF and other titinopathies but in different muscles. The central pathway focuses on myofibrillar myopathies, characterized by sparing or late involvement of the semitendinosus muscle. The right pathway is tailored for GNE myopathy, emphasizing early semitendinosus involvement, other posterior thigh muscle involvement, early gastrocnemius and soleus involvement, and marked quadriceps sparing.

**
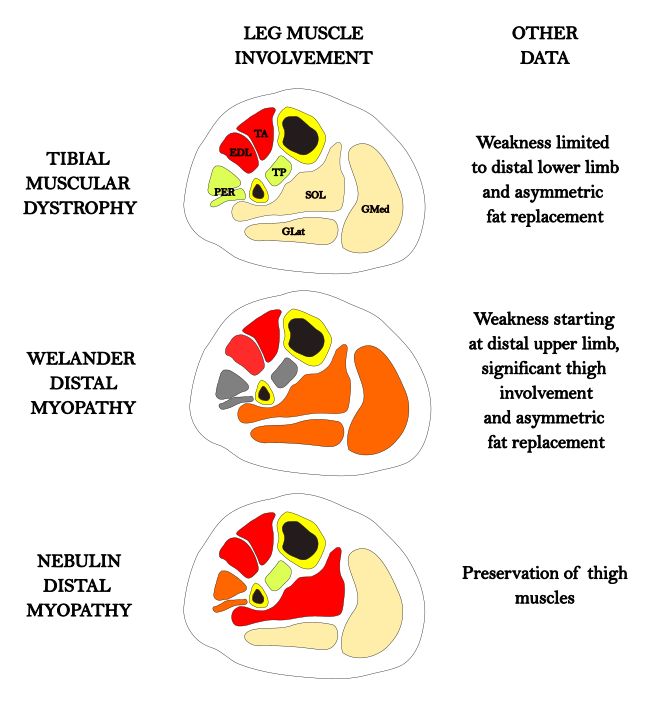
**

**Supplementary figure 5**. **Patterns of muscle involvement in distal myopathies which are a diffential diagnosis for tibial muscular dystrophy.** Cross-sectional illustrations of the leg vdemonstrate the distribution of muscle involvement in three distal myopathies: Tibial Muscular Dystrophy (TMD), Welander Distal Myopathy, and Nebulin Distal Myopathy. The colored areas within the muscle compartments indicate the probability of fat replacement, with a color gradient. Muscles in red are nearly always involved, muscle in dark oragns are commonly involved, muscles in light orange are sometimes involved and muscles in green are usually preserved. Grey muscles are those in which we do not have clear data. For each condition, the right column describes additional clinical or radiological characteristics. Abbreviations: TA tibialis anterior, EDL extensor digitorum longus, PER peroneal muscles, TP: tibialis posterior, SOL soleus, GLat gastrocnemius lateralis and GMed gastrocnemius medialis.
